# Draft genome sequence of *Nitratireductor* sp. PBL-C9—a marine bacterium encoding glycoside hydrolases isolated from Batu Laut Beach in Selangor, Malaysia

**DOI:** 10.1128/mra.00683-25

**Published:** 2025-08-11

**Authors:** Ummirul Mukminin Kahar, Nasibah Abdul Rahman, Nabila Aisyah Sharoul, Farzana Muhammad Ramzan

**Affiliations:** 1 Malaysia Genome and Vaccine Institute, National Institutes of Biotechnology Malaysia, Kajang, Selangor, Malaysia; 2 Faculty of Science and Technology, Universiti Malaysia Sabah 60606, Kota Kinabalu, Sabah, Malaysia; 3 Department of Plant Science, Kulliyyah of Science, International Islamic University Malaysia 162083, Kuantan, Pahang, Malaysia; 4 Faculty of Science and Marine Environment, Universiti Malaysia Terengganu 95438, Kuala Terengganu, Terengganu, Malaysia; Indiana University, Bloomington, Bloomington, Indiana, USA

**Keywords:** *Nitratireductor*, genome, Batu Laut Beach, marine bacteria, carbohydrate degrading enzyme, glycoside hydrolase, α-amylase, β-glucosidase, starch, cellulose

## Abstract

This study presents a high-quality annotated draft genome of *Nitratireductor* sp. PBL-C9—a marine bacterium isolated from Batu Laut Beach in Selangor, Malaysia. The genome encoded α-amylase and β-glucosidase, suggesting potential applications in the food and biofuel industries. These findings provide new insights into the biotechnological prospects of *Nitratireductor* spp.

## ANNOUNCEMENT

*Nitratireductor* spp. are halotolerant or halophilic bacteria predominantly found in marine environments (1). Previous studies have identified several enzymes in this genus (2–4); however, glycoside hydrolases (GHs) have not been reported.

*Nitratireductor* sp. PBL-C9 was isolated from wet sediment and mud (top 15 cm layer) collected from Batu Laut Beach in Selangor, Malaysia (2.6747°N, 101.5166°E), using an *ex situ* cultivation technique (5). A 3 L diffusion bioreactor was used, with the inner chamber containing 300 mL of sterilized marine broth (Condalab, Spain) and the outer chamber filled with the environmental samples. After 2 weeks of incubation at 30°C, 1.0 mL of the enriched culture was aseptically transferred into 9.0 mL of 0.5% (wt/vol) NaCl solution. Serial dilutions (10⁰–10⁻¹²) were plated on marine agar and incubated at 30°C for 14 days. Pure colonies of PBL-C9 were obtained by streaking the cells on marine agar (pH 8.0) and incubating at 30°C for 72 h. Genomic DNA was extracted using the Monarch Genomic DNA Purification Kit (NEB, USA), according to the manufacturer’s instructions. The 16S rRNA gene was amplified using primers 27F and 1492R (6), sequenced, and taxonomically identified using NCBI BLASTn and the EzBioCloud 16S database (7). The analyses revealed that PBL-C9 was most closely related (99.57%) to *Nitratireductor aquimarinus* CL-SC21^T^ (GenBank accession number HQ176467.1).

PBL-C9 was grown on marine agar (pH 8.0) at 30°C for 72 h. Genomic DNA was extracted from a single colony of the cells according to the aforementioned method. A paired-end library was prepared using the standard protocol of the NEBNext Ultra II DNA Library Preparation Kit for Illumina (New England BioLabs, USA). Sequencing was performed using the NovaSeq X system with 150 bp paired-end reads (Illumina, USA). The raw reads were trimmed using Fastp v0.24.1 (8), *de novo* assembled using SOAPdenovo v2.04 (9), and annotated using the NCBI Prokaryotic Genome Annotation Pipeline v6.10 (10). Genome comparison between PBL-C9 and all 256 available genomes of *Nitratireductor* spp. in the NCBI genome database (June 2025) was performed using digital DNA–DNA hybridization (dDDH) via the Genome-to-Genome Distance Calculator (GGDC) v3.0 (11) and the average nucleotide identity (ANI) function in the EzBioCloud server (12). Carbohydrate-active enzymes (CAZymes) were mined using dbCAN3 (13). Default parameters were used for all software tools, unless otherwise specified.

The sequencing produced 1,480,716,900 bases from 4,935,723 paired-end reads. The assembled genome size was 4,794,337 bp in 12 contigs, with 307× coverage, an N_50_ of 408,338 bp, and a G + C content of 62.0%. Genome annotation identified 4,514 genes, comprising 4,438 protein-coding genes, 58 RNAs (each one of 5S, 16S, and 23S rRNA, 51 tRNAs, and 4 ncRNAs), and 18 pseudogenes. Genome comparative analyses showed that PBL-C9 is closely related to *N. aquimarinus* F7 (dDDH, 88.80%; ANI, 98.68%) (Table 1). The PBL-C9 genome encoded 78 CAZymes, including 32 GHs, 28 glycosyltransferases, 11 auxiliary activity enzymes, and 7 carbohydrate esterases. Notably, genes encoding for GH13 α-amylase and GH1 β-glucosidase were identified—key enzymes in food and biofuel production, respectively (14, 15). In summary, the PBL-C9 genome provides GH candidates for industrial applications.

**TABLE 1:** Genomic comparison of strain PBL-C9 against all 256 available genomes of *Nitratireductor* spp. using dDDH and ANI

Strain	Value between strain PBL-C9 and other *Nitratireductor* genomes	
	dDDH (%)	ANI (%)
*Nitratireductor aquimarinus* F7	88.80	98.68
*Nitratireductor *sp. ECS34B2	88.70	98.67
*Nitratireductor aquimarinus* EA12-06	88.70	98.67
*Nitratireductor aquimarinus* ES21-04	88.60	98.63
*Nitratireductor* sp. DP7N14-4	88.60	98.63
*Nitratireductor aquimarinus* SY-2-4	88.60	98.60
*Nitratireductor aquimarinus* ET19-04	88.40	98.62
*Nitratireductor aquimarinus* EA08-01	88.40	98.59
*Nitratireductor aquimarinus* TN35-13	88.30	98.61
*Nitratireductor aquimarinus* TN21-10	88.30	98.57
Remaining 246 genomes of *Nitratireductor *spp.	17.30–88.20	65.53–84.07

## Data Availability

This Whole Genome Shotgun project has been deposited in NCBI GenBank under the BioProject accession number PRJNA1271266, BioSample accession number SAMN48859005, and GenBank accession number JBPAXW010000000. The version described in this paper is the first version (JBPAXW000000000.1). The raw sequencing reads have been deposited in the NCBI Sequence Read Archive (SRA) under the accession number SRR33822578. The 16S rRNA gene sequence of *Nitratireductor* sp. PBL-C9 has been deposited in NCBI GenBank under the accession number PV731398.1.
